# Bacterial surface‐exposed lipoproteins and sortase‐mediated anchored cell surface proteins in plant infection

**DOI:** 10.1002/mbo3.1382

**Published:** 2023-09-14

**Authors:** Andrés de Sandozequi, Claudia Martínez‐Anaya

**Affiliations:** ^1^ Departamento de Ingeniería Celular y Biocatálisis Instituto de Biotecnología Cuernavaca México

**Keywords:** bacterial cell surface, lipoprotein, phytopathogenic bacteria, sortase, surfaceome, surface proteins

## Abstract

The bacterial cell envelope is involved in all stages of infection and the study of its components and structures is important to understand how bacteria interact with the extracellular milieu. Thanks to new techniques that focus on identifying bacterial surface proteins, we now better understand the specific components involved in host–pathogen interactions. In the fight against the deleterious effects of pathogenic bacteria, bacterial surface proteins (at the cell envelope) are important targets as they play crucial roles in the colonization and infection of host tissues. These surface proteins serve functions such as protection, secretion, biofilm formation, nutrient intake, metabolism, and virulence. Bacteria use different mechanisms to associate proteins to the cell surface via posttranslational modification, such as the addition of a lipid moiety to create lipoproteins and attachment to the peptidoglycan layer by sortases. In this review, we focus on these types of proteins (and provide examples of others) that are associated with the bacterial cell envelope by posttranslational modifications and their roles in plant infection.

## Graphical abstract

We reviewed the literature on bacterial cell surface lipoproteins and proteins attached to the peptidoglycan layer and their importance in plant‐bacteria interactions.

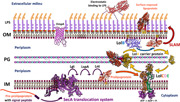

## 1 INTRODUCTION

**Figure 1:**
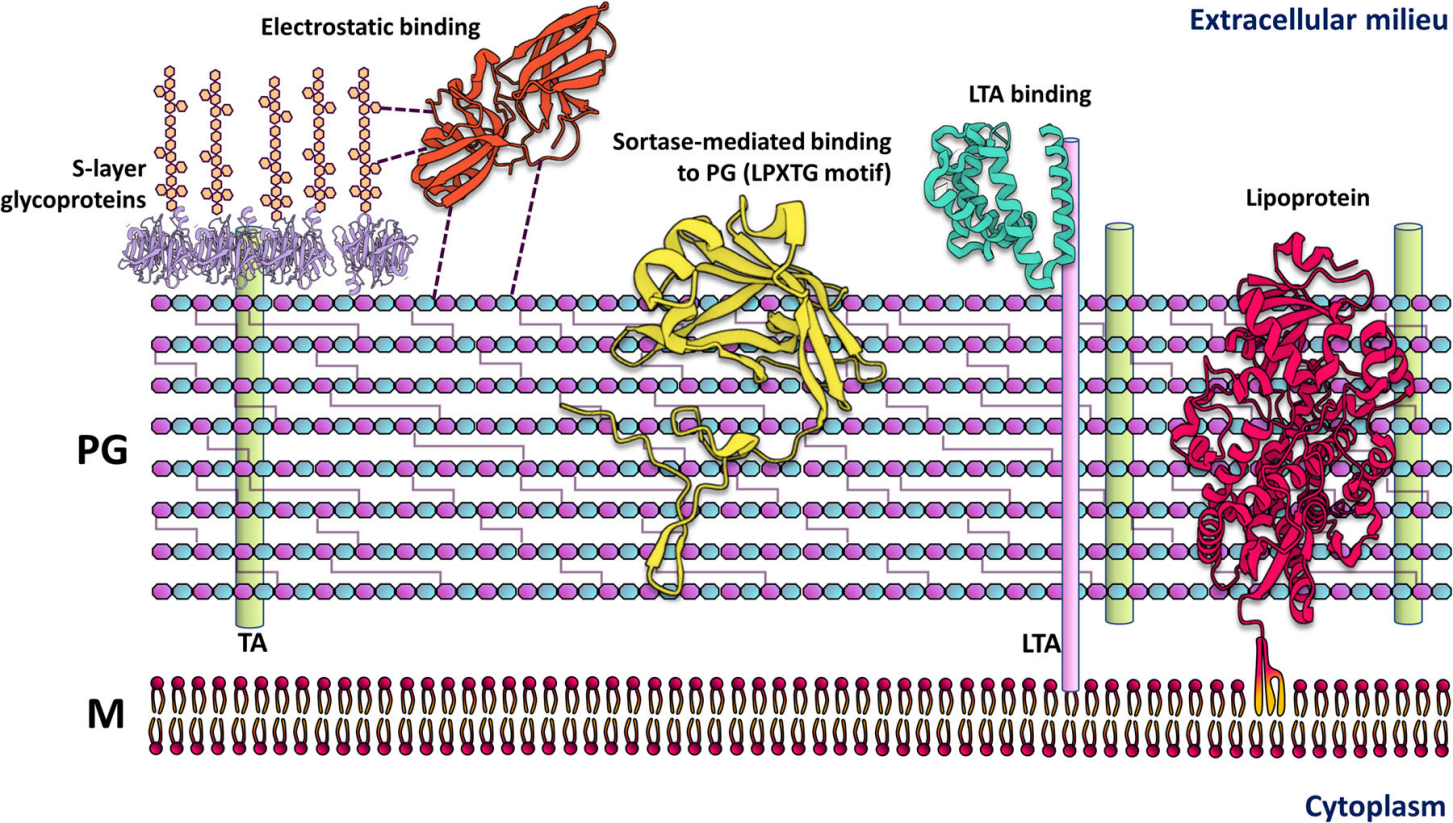
Surface‐exposed proteins in monoderm bacteria. In monoderms, proteins attach to the surface through noncovalent direct binding or binding by specialized protein domains and electrostatic interactions to cell wall components such as the peptidoglycan (PG) layer and lipoteichoic acids (LTA) (Chagnot et al., 2013; Desvaux et al., 2006, 2018). Another mechanism involves the covalent binding of proteins with an LPXTG motif in their C‐terminal region by sortases. The LPXTG motif is cleaved between threonine and glycine residues, and sortases attach the target protein to the Lipid II molecule, which is the building block of the growing PG layer, specifically at the Thr residue. (Dai et al., 2019; Desvaux et al., 2018; Paterson & Mitchell, 2004). In the case of monoderm lipoproteins depending on their size or other characteristics, they can span the PG layer to be surface exposed (this is the case of some adhesins and surface enzymes, for instance [Fischetti, 2019]). M, membrane; TA, teichoic acid.

**Figure 2:**
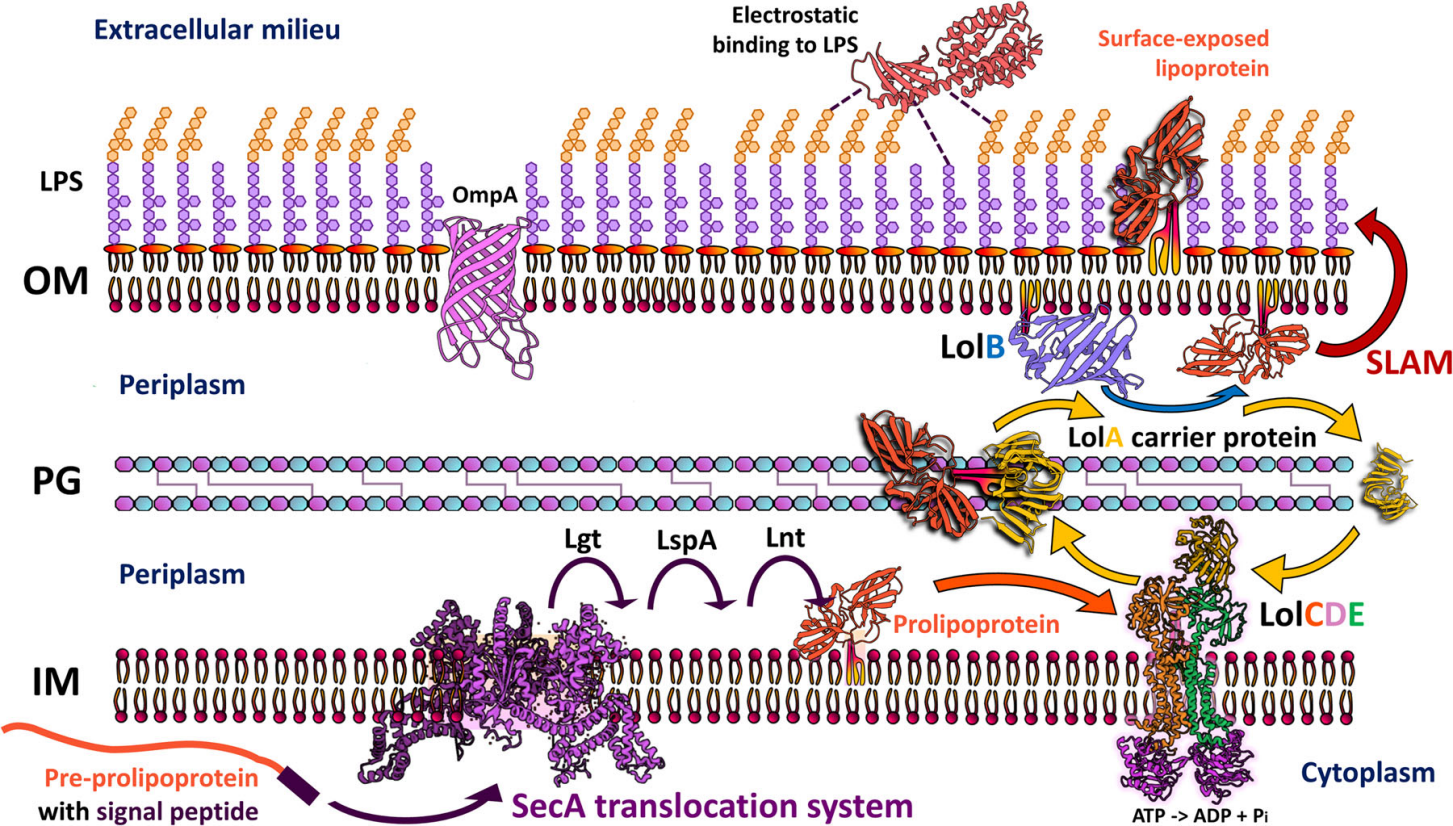
Surface‐exposed proteins in diderm bacteria. In diderms, after translocation by the Sec or Tat systems, and posttranslational modification by the lipidation machinery (Lgt, LspA, and Lnt), the LolABCDE system correctly sorts and localizes lipoproteins. The LolCDE complex detaches the prolipoprotein from the inner membrane using ATP and transfers the LolA carrier protein. LolA then transports the lipoprotein across the periplasm and delivers it to LolB to be anchored to the inner side of the outer membrane. These lipoproteins can then be transposed to the extracellular side of the outer membrane by the SLAM or Bam complexes to become OM lipoproteins (Cole et al., 2021; Hooda & Moraes, 2018; Wilson & Bernstein, 2016). Other surface‐exposed proteins can be embedded in the OM by transmembrane or pore‐forming domains, such as those of the outer‐membrane protein A (OmpA) family. Some proteins establish electrostatic interactions with lipopolysaccharide (LPS) chains in the outer membrane. IM, inner membrane; OM, outer membrane; PG, peptidoglycan.

Bacterial infectious disease progression involves a stage of surface colonization during which cells adapt and produce proteins involved in pathogenesis. Technological advances in the analysis of the bacterial surfaceome and proteosurfaceome (Desvaux et al., 2018; Rodríguez‐Ortega, 2018), have facilitated identifying proteins relevant to virulence and infection, yet their importance extends beyond characterizing these processes as they are potential drug targets and candidates for protein‐based vaccines. In the context of bacterial phytopathogens, surface proteins have been somewhat overlooked, as most of them are not considered effectors, but understanding them could lead to breakthroughs in the fight against phytopathogenic bacteria. The bacterial cell envelope is the first structure to interact with plant tissues, and it often serves as the furthermost line of attack to colonize and infect plant hosts. The cell envelope is composed of a variety of molecules, including polysaccharides, lipids, and proteins (Bogino et al., 2013; Silhavy et al., 2010; Viljoen et al., 2020), and of these, the proteins have numerous functions in protection, secretion, biofilm formation, nutrient intake, metabolism, and virulence. Bacteria posttranslationally modify proteins to associate them to the cell surface, for instance, during extracellular trafficking by sortases (Figure 1) (Dai et al., 2019); also, by adding a lipid moiety to create membrane‐bound lipoproteins (Figure 2) (Cole et al., 2021; Kovacs‐Simon et al., 2011; Narita & Tokuda, 2017). Numerous mechanisms for binding proteins to the bacterial surface have been identified and extensively documented in the literature (Fischetti, 2019). Still, novel mechanisms are being discovered and are in the process of being comprehensively understood. These include: the attachment of a lipopolysaccharide A (A‐LPS) by the type XI secretion system carried out in the superphylum fibrobacteres–chlorobi–bacteroidetes (De Diego et al., 2016; Veith et al., 2017); attachment to the extracellular matrix (e.g., to the S‐layer in monoderm bacteria; Figure 1) (Desvaux et al., 2006; Flemming & Wingender, 2010); and binding to other surface components due to the presence of protein domains, or embedding into membranes (for instance: adhesins, pore‐forming domains, cell wall binding domains, and transmembrane helices, Figure 2) (Bateman et al., 2021; Carter et al., 2023; Desvaux et al., 2018; Mhedbi‐Hajri et al., 2011; Rodríguez‐Ortega, 2018; Vergalli et al., 2020). In phytopathogenic bacteria, surface‐associated proteins accomplish physiological and invasive roles (Cole et al., 2021), but different studies show the involvement of the bacterial surface in pathogenesis (Bogino et al., 2013; Desvaux et al., 2018; Pfeilmeier et al., 2016; Rodríguez‐Ortega, 2018). Although these studies are mainly centered on human pathogens, interest has increased in understanding how the bacterial surface is involved in the process of infection by plant pathogenic bacteria.

## 2 SURFACE‐EXPOSED PROTEINS ANCHORED TO THE PEPTIDOGLYCAN LAYER

Monoderm bacteria contain only one lipid bilayer and a thick peptidoglycan layer. In contrast to diderm bacteria that have two membranes and a peptidoglycan layer in between, in monoderms, the peptidoglycan layer is the outermost structure interacting with the exterior, and there, surface proteins play important roles. Monoderms attach proteins to the peptidoglycan layer in a unique manner via the sortase system. Sortases are peptidoglycan‐bound enzymes that recognize a motif in the C‐terminal region of target proteins. The most studied is the LPXTG motif, which is recognized by sortases conserved across multiple bacterial clades. Sortases recognize and cleave this motif between Thr and Gly residues and anchor the LPXTG‐containing protein to the lipid II molecule, which is the building block of the growing peptidoglycan layer (Comfort & Clubb, 2004; Dai et al., 2019; Desvaux et al., 2006; Dramsi et al., 2008; Hendrickx et al., 2011; Navarre & Schneewind, 1994; Novick, 2000; Pishesha et al., 2018). Indeed, sortase‐mediated anchoring of surface proteins is important for some bacterial pathogens such as staphylococci, streptococci, enterococci, and *Listeria monocytogenes* (Dai et al., 2019; Egan et al., 2010; Hendrickx et al., 2009, 2011). In *Clavibacter michiganensis* ssp. *michiganensis*, an important pathogen of tomatoes and potatoes, sortase SrtA is essential for blister formation, which is a hallmark of the infectious process, but not for wilting symptoms, suggesting that it is important for the attachment of bacteria to the leaf surface (Chalupowicz et al., 2017). Furthermore, an important virulence factor of this pathogen, the cell wall‐bound protease Pat‐1—a substrate of SrtA—is absent in some nonvirulent isolates of *Clavibacter* species (Gartemann et al., 2003). In *Bacillus* (many of which are plant growth‐promoting bacteria and in some cases opportunistic pathogens), sortases are vital for plant colonization and are involved in pili and endospore biosynthesis (Budzik et al., 2007). Inhibition of sortases as a therapeutic target in multidrug resistant pathogens has been a hot topic (Cascioferro et al., 2014; Cossart & Jonquières, 2000; Kudryavtsev et al., 2021; Ouyang et al., 2018; Si et al., 2016), and could prove useful in dealing with phytopathogenic bacteria, but more research is needed to fully understand all the implications that sortases and their substrates have for plant disease. Interestingly, expansin EXLX1 from *Bacillus subtilis* is required for colonizing maize roots (Kerff et al., 2008), and although it binds insoluble peptidoglycan with high affinity in vitro, its sequence contains a sortase motif followed by a hydrophobic region (de Sandozequi et al., 2022), strongly suggesting it to be a substrate for a sortase. Furthermore, other expansins from related bacteria also contain putative sortase‐recognition signatures, some of which are plant pathogens such as *Bacillus pumilus, Streptomyces scabies*, and *Streptomyces ipomoeae* (de Sandozequi et al., 2022).

## 3 LIPOPROTEINS IN PLANT‐PATHOGENIC BACTERIA

Diderm and monoderm bacteria produce lipoproteins, being the periplasm the predominant localization site for most lipoproteins in diderms (Figures 1 and 2). Due to the identification of the outer membrane protein translocons, SLAM and Bam, in recent years, there has been an increased discovery of surface‐exposed lipoproteins that are embedded in the periplasmatic side of the outer membrane and exposed to the surface (Figure 2) (El Rayes et al., 2021; Huynh et al., 2022; Kovacs‐Simon et al., 2011; Remans et al., 2010; Wilson & Bernstein, 2016), which leads us to question the implications of these proteins in cell function. For instance, more than half of the 175 predicted lipoproteins in the genome of *Pseudomonas aeruginosa* are classified as hypothetical proteins for which a function, and thus their involvement in pathogenesis, remains to be determined (Nguyen et al., 2020; Remans et al., 2010). Similar numbers were predicted (at around 2% of total lipoprotein genes per genome) (Babu et al., 2006; Teufel et al., 2022) for the most important plant pathogens (Mansfield et al., 2012). As our knowledge of lipoprotein signal peptides grows and more accurate algorithms for their prediction are developed, we will continue finding more lipoproteins that are essential to bacterial physiology, but potentially, also for the interaction with their hosts (Table 1). In comparison, the DOLOP database, which was the first comprehensive database for lipoproteins, had certain limitations in recognizing lipoprotein signal peptides across various bacterial species (Madan Babu & Sankaran, 2002; Babu et al., 2006). More available genetic information and knowledge of the properties of signal peptides have led to the development of better software for their analysis. The most advanced algorithm so far that predicts all known types of signal peptides (including those not yet known when the DOLOP database was created), is SignalP which is currently in its 6.0 version. SignalP v6.0 has introduced a novel machine‐learning method that recognizes and classifies five different types of signal peptides, and it can predict signal peptides in distantly related sequences or metagenomic sequences from unknown origin (Almagro Armenteros et al., 2019; Teufel et al., 2022), which could help to identify potential lipoproteins from newly discovered or unculturable plant‐pathogens that cannot be studied in vitro.

**Table 1:** The number of predicted lipoprotein genes in important phytopathogens.

Genome	DOLOP database^a^	By using SignalP 6.0^b^
*Agrobacterium fabrum* C58 (*Agrobacterium tumefaciens* strain C58)	29	65
*Burkholderia pseudomallei* K96243	93	209
*Pectobacterium atrosepticum* SCRI1043	112	142
*Pectobacterium brasiliense* BC1	N/P	125^c^
*Pectobacterium carotovorum* subsp. *carotovorum*	N/P	159^c^
*Pseudomonas aeruginosa* PAO1	113	204
*Pseudomonas putida* KT2440	74	166
*Pseudomonas syringae* pv. *tomato* DC3000	101	173
*Ralstonia solanacearum* GMI1000	47	191
*Streptomyces scabiei* (strain 87.22)	N/P	238
*Xanthomonas axonopodis* pv. *citri* 306	92	171^c^
*Xanthomonas albilineans* GPE PC73	N/P	131
*Xanthomonas campestris* pv. *campestris* ATCC 33913	95	186
*Xanthomonas oryzae* pv. *oryzae* KACC10331	58	122
*Xylella fastidiosa* 9a5c	47	79

^a^ DOLOP database (Babu et al., 2006).

^b^ Lipoprotein genes predicted using SignalP 6.0 (Teufel et al., 2022) in reference proteomes from UniProt release 2021_02; these include signal peptides for Sec/SPII and Tat/SPII processing.

^c^ For important phytopathogen without a reference proteome, SignalP 6.0 online server (https://services.healthtech.dtu.dk/service.php?SignalP-6.0) was used to predict the number of lipoproteins. N/P, species absent in the DOLOP database.

Lipoproteins are involved in processes such as maintaining bacteria cell envelope integrity (Dubuisson et al., 2005; Hellman et al., 2002; Lazzaroni & Portalier, 1992), cellular morphology (Paulsson et al., 2021), drug efflux (Li et al., 1995), and iron uptake and protein secretion (Alvarez‐Martinez et al., 2021; El Rayes et al., 2021; Green & Mecsas, 2016). Some lipoproteins are part of cellular appendages such as flagella and pili (Jin et al., 2001), while others are integral components of the peptidoglycan biosynthesis machinery and transport systems for effectors, proteins, lipopolysaccharides, and antibiotics (Lorenz et al., 2019; Okuda & Tokuda, 2011). In some cases, lipoproteins are enzymes that have proteolytic and plant cell wall‐degrading activities (Liao et al., 2022). Besides the roles described for lipoproteins in model bacteria and human pathogens, phytopathogenic bacteria often rely on lipoproteins to attach to their preferred biological substrates, which is an important step in host colonization. The LolABCDE system (Figure 2) plays a critical step in the correct sorting of periplasmic and surface‐exposed lipoproteins in phytopathogens (Konovalova et al., 2017; Narita & Tokuda, 2017; Okuda & Tokuda, 2011; Wilson & Bernstein, 2016). A particular component of this system is the LolB lipoprotein, which tethers the target lipoprotein to the outer lipid bilayer (Hooda & Moraes, 2018; Wilson & Bernstein, 2016). A recent study showed that a *Xanthomonas campestris* pv. *campestris* mutant strain unable to produce LolB has pleiotropic phenotypes, ranging from decreased biofilm production to decreased virulence. LolB is required for the correct attachment of lipoproteins to the outer layer, so these symptoms could be explained by the general defect in cell membrane integrity and the perturbation of lipoprotein homeostasis (Liao et al., 2022). LolB is also required for *in planta* survival in the xylem‐invading phytopathogen *Ralstonia solanacearum* (Su et al., 2021).

Lipoproteins with unusual functions exist in phytopathogenic bacteria. For example, the two surface‐exposed small lipoproteins EcnA and EcnB (originally described as components of the entericidin toxin‐antitoxin system in an *Enterobacter* strain (Bishop et al., 1998; Schubiger et al., 2015) also modulate cell aggregation, biofilm formation, motility, outer membrane vesicle release, and resistance to reactive oxygen species in *Xanthomonas cirti* subsp. *citri* and *Agrobacterium tumefaciens* (Granato et al., 2019; Knoke et al., 2020; Sidhu et al., 2008). Lipoproteins also participate in host–pathogen interactions. For example, the lipoprotein VacJ is a component of the lipid asymmetry maintenance machinery, and when absent in *X. citri* results in decreased biofilm formation in glass tubes and on leaf surfaces, decreased swarming motility, reduced bacterial growth in planta, and reduced virulence (Li & Yu, 2020). Another lipoprotein of *X. citri*, OmlA, is important for multidrug resistance, and it may be implicated in protein–protein interactions and maintenance of the outer membrane integrity (Fuangthong et al., 2008; Vanini et al., 2008). Lipoproteins are also important for the production and export of exopolysaccharides in some bacteria, for example, the lipoprotein GumB is involved in the biosynthesis of xanthan gum by *X. campestris* (Jacobs et al., 2012). In *Rhizobium leguminosarum*, the lipoprotein PssN is part of the exopolysaccharide polymerization and export complex PssTNOP, which are needed for the successful invasion of its host (Marczak et al., 2006; Wielbo et al., 2004). In a previous report by our laboratory, we found that in diderm bacteria a third of the expansin precursors have a lipoprotein signal peptide, principally in the phytopathogenic members of Burkholderiales (excluding *Ralsontia* species) and Xanthomonadales, in the soil‐dwelling Myxococcales, and in the Gram‐positive Frankiales order (de Sandozequi et al., 2022). In the Fibrobacteres–Chlorobi–Bacteroidetes (FCB) superphylum, expansins contain a C‐terminal domain recognized by the recently discovered type 9 secretion system that attaches A‐LPS to the protein (Veith et al., 2017), and Cryptosporangiales expansins contain a transmembrane domain (de Sandozequi et al., 2022). The biological function and mechanism of action of bacterial expansins have yet to be determined, but it is likely associated with plant cell wall degradation and virulence. Several reports of expansin mutants from plant growth‐promoting and phytopathogenic bacteria evidence a role for expansins during colonization and infection of the plant host (Georgelis et al., 2011; Junior et al., 2015; Narváez‐Barragán et al., 2020; Pastor et al., 2015; Tancos et al., 2018). Although in monoderms, most lipoproteins attach to the outer layer of the cytoplasmic membrane and are covered by the peptidoglycan layer (Figure 1), some lipoproteins could be big enough or have specialized domains that allow them to be exposed to the surface (Fischetti, 2019; Nguyen et al., 2020; Saleh et al., 2013), but knowledge of lipoprotein involvement in plant pathogenesis by monoderm bacteria is still lacking. Nevertheless, lipoproteins in monoderm bacteria are needed for general housekeeping and cell wall homeostasis, therefore important for the survival of bacteria in any environment (Desvaux et al., 2018; Nguyen et al., 2020).

## 4 PERSPECTIVES

Surface‐associated proteins participate in plant colonization and infection by bacteria, and some are important in phytopathogenic bacterial fitness. For example, gene RSc2007, encoding an unknown lipoprotein (UniProt Id: Q8XXV6), appears to be a xylem sap fitness factor in *R. solanacearum* (Georgoulis et al., 2021). Importantly, lipoproteins could be the target of potential biocontrol targets, such as the coumarins that inhibit the expression of the outer membrane lipoprotein OmlA in *X. campestris* (Fuangthong et al., 2008). Yet, a large number of predicted lipoproteins and cell wall‐anchored proteins with unknown functions still need characterization bringing about new possibilities for identifying bacteria‐plant interactors, some of which may be key players for virulence. Finally, we must leverage artificial intelligence and machine learning tools (such as SignalP v6.0 [Teufel et al., 2022], CW‐PRED (Fimereli et al., 2012), and AlphaFold (Jumper et al., 2021) for faster and more precise identification of surface‐associated proteins in newly sequenced genomes of emerging and established phytopathogens as agriculture crises require diverse strategies to combat the challenges at hand.

## AUTHOR CONTRIBUTIONS

**Andrés de Sandozequi**: Conceptualization (equal); data curation (equal); writing—original draft (lead); writing—review and editing (equal). **Claudia Martínez‐Anaya**: Conceptualization (equal); data curation (equal); funding acquisition (equal); project administration (equal); writing—original draft (supporting); writing—review & editing (equal).

## CONFLICT OF INTEREST STATEMENT

None declared.

## ETHICS STATEMENT

None required.

## Data Availability

Not applicable.
